# Proof of Concept of the Ability of the Kinect to Quantify Upper Extremity Function in Dystrophinopathy

**DOI:** 10.1371/currents.md.9ab5d872bbb944c6035c9f9bfd314ee2

**Published:** 2013-03-14

**Authors:** Linda P Lowes, Lindsay N Alfano, Brent A Yetter, Lise Worthen-Chaudhari, William Hinchman, Jordan Savage, Patrick Samona, Kevin M Flanigan, Jerry R Mendell

**Affiliations:** Nationwide Children’s Hospital, Columbus, Ohio, United States; Center for Gene Therapy, The Research Institute at Nationwide Children's Hospital, Columbus, Ohio, United States; The Research Institute at Nationwide Children’s Hospital, Columbus, Ohio, United States; Department of Physical Medicine and Rehabilitation & Center for Personalized Health Care, The Ohio State University, Columbus, Ohio, United States; Vectorform, Royal Oak, Michigan, United States; Vectorform, Royal Oak, Michigan, United States; Vectorform, Royal Oak, Michigan, United States; Departments of Neurology and Pediatrics, The Ohio State University, Columbus, Ohio, United States; Center for Gene Therapy, Nationwide Children's Hospital, Columbus, Ohio, United States; Departments of Neurology and Pediatrics, The Ohio State University, Columbus, Ohio, United States; Center for Gene Therapy, Nationwide Children's Hospital, Columbus, Ohio, United States

## Abstract

Introduction: Individuals with dystrophinopathy lose upper extremity strength in proximal muscles followed by those more distal. Current upper extremity evaluation tools fail to fully capture changes in upper extremity strength and function across the disease spectrum as they tend to focus solely on distal ability. The Kinect by Microsoft is a gaming interface that can gather positional information about an individual’s upper extremity movement which can be used to determine functional reaching volume, velocity of movement, and rate of fatigue while playing an engaging video game. The purpose of this study was to determine the feasibility of using the Kinect platform to assess upper extremity function in individuals with dystrophinopathy across the spectrum of abilities. Methods: Investigators developed a proof-of-concept device, ACTIVE (Abilities Captured Through Interactive Video Evaluation), to measure functional reaching volume, movement velocity, and rate of fatigue. Five subjects with dystrophinopathy and 5 normal controls were tested using ACTIVE during one testing session. A single subject with dystrophinopathy was simultaneously tested with ACTIVE and a marker-based motion analysis system to establish preliminary validity of measurements. Results: ACTIVE proof-of-concept ranked the upper extremity abilities of subjects with dystrophinopathy by Brooke score, and also differentiated them from performance of normal controls for the functional reaching volume and velocity tests. Preliminary test-retest reliability of the ACTIVE for 2 sequential trials was excellent for functional reaching volume (ICC=0.986, p<0.001) and velocity trials (ICC=0.963, p<0.001). Discussion: The data from our pilot study with ACTIVE proof-of-concept demonstrates that newly available gaming technology has potential to be used to create a low-cost, widely-accessible and functional upper extremity outcome measure for use with children and adults with dystrophinopathy.

## Introduction

Individuals with dystrophinopathy undergo progressive loss of muscle strength and function across the lifespan 1 ^,^ 2 . This loss of function impacts independence in ambulation, self-care, and other daily activities directly influencing quality of life 3 ^,^ 4. For the first time in decades there are potential treatments on the horizon for individuals with Duchenne muscular dystrophy (DMD) through exon skipping, small molecules and gene therapy^5^. Although these trials are providing hope for patients with DMD for the very first time, they are not accessible to all individuals with this disease. These experimental trials use timed walking as the primary functional outcome measure due to ease of test administration, ability to quantify distance walked, and literature investigating the reliability and validity of these walking outcomes^6^^-^ 8. Boys who cannot walk are therefore not eligible for participation in these initial pivotal trials. The biggest obstacle to performing clinical trials that address function in the boys and men who are non-ambulatory is the dearth of adequate, quantifiable, and sensitive upper extremity (UE) outcome measures to capture changes over time^9^. This leaves an enormous unmet need for a large proportion of persons with neuromuscular disease who can no longer walk; and therefore given delayed access to these potentially lifesaving drugs due to the lack of a valid and reliable UE functional outcome measure.

Dystrophinopathy causes a characteristic loss of muscle strength in proximal muscles first followed by distal musculature over time. Current outcome measures do not accurately measure changes in strength and function that occurs in individuals with dystrophinopathy across the lifespan 10. There are many reasons for this. First, these outcomes, such as the Jebsen Taylor Hand Function Test, 9 Hole Peg Test, Melbourne Assessment, were originally designed for other patient groups and do not capture the unique weakness patterns found in dystrophinopathy 9 ^,^ 11 ^-^ 17. Despite the integral interdependence of both proximal and distal muscles necessary for function, many current UE outcome measures typically focus on distal abilities alone or include limited evaluation of anti-gravity movements requiring proximal muscle strength 9 ^,^ 10 ^,^ 14 ^-^ 16. Patient reported outcome measures such as the Egan Klassifikation scale provide information regarding an individual’s overall level of functioning, but are too broad to measure change in clinical trials 9 ^,^ 18 ^,^ 19. Lastly, many of these functional outcomes use an ordinal level Likert scale that limits statistical analysis. A continuous scale outcome measure designed to measure full UE abilities over time in patients with dystrophinopathy is in critical need at this time of ground-breaking clinical trials.

Video-based assessments have the potential to accurately collect data, removing some of the examiner-rater bias of existing scales. The Microsoft Kinect is a controller-free gaming device interface that can document participant motion, including the full UE from shoulder to hand. The Kinect is able to track participant motion using the imbedded infrared camera to record positional data over time. Through the capture of participant motion, the Kinect has the potential to gather information related to functional reaching volume (FRV), velocity of movement, and rate of fatigue. Functional reaching volume is the space within which a person can interact with the environment around them 20 ^-^ 22. Measurement of this variable, would allow researchers to quantify functional ability. A larger FRV would likely correlate with an increase in functional ability 20 ^-^ 22. Velocity of reaching is a second component of UE function necessary for independence and is important because, if activity performance is too slow and cumbersome, a caregiver will likely take over the task and an area of independence is lost 23. One final movement component essential to independent function is the ability to sustain movement. The rate in which a person fatigues, or the rate in which their velocity declines, is directly related to the amount of functional work or play that can be performed in a day 24 ^-^ 26. A person’s rate of fatigue dictates whether they can participate in leisure activities, attend school or hold a job 27. The Kinect framework also has the potential to utilize gaming to increase participant motivation over time to reliably measure full UE abilities.

In collaboration with a software development company we conducted a feasibility study to determine if the Kinect platform could be used to create an UE outcome measure for dystrophinopathy. An assessment tool was developed to measure UE functional reaching volume, velocity, and rate of fatigue and a small pilot study was completed to establish proof of concept.

## Methods

This proof-of-concept called the ACTIVE (Ability Captured Through Interactive Video Evaluation) used the Microsoft Kinect for Windows controller-free gaming interface to capture movement of the UE while a subject plays 2 custom-designed video games (Figure 1). The Kinect relies on its built-in video camera and 3-dimensional (3D) depth sensors (an active-sensing depth camera using structured light) to capture and record participant motion. The Kinect projects an infrared reference matrix that is offset by and reflects off of the person in front of the system. This is then captured by a depth sensor to generate a high-resolution depth map of each pixel allowing for tracking of movement. Current estimates of resolution at 2 meters from the Kinect system are 3 mm in the horizontal and vertical axes and 10 mm in depth 28.

**Figure:**
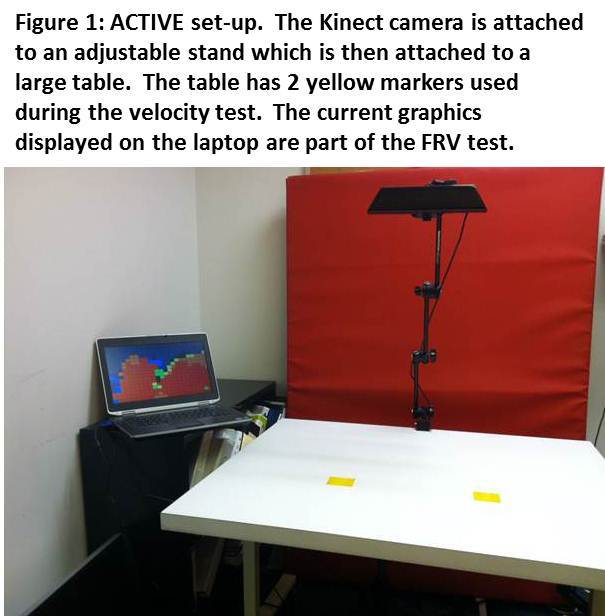

Computations for creating the depth map were handled through the PrimeSense image processor chip embedded within the Kinect device. The ACTIVE software calculated depth from disparity of the projected reference matrix to the captured offset matrix. In addition to the depth sensor; the Kinect has an RGB (red, green, and blue) camera based on a complementary metal-oxide semiconductor that delivers the three basic color components that can be combined to provide a full color image. Using these data, the ACTIVE software calculated the centroid of a contrasting color sticker that had been placed on the subject’s hand. Using a combination of the depth and color camera systems, the ACTIVE software measured participants’ functional reaching volume, velocity of movement, and rate of fatigue.

To capture these variables, two basic games were created. ACTIVE’s FRV game used the Kinect’s 3D depth sensor to determine the space or envelope through which a player reached during a virtual coloring game. The subject was encouraged to reach as far as possible in every direction to fully paint the screen. ACTIVE calculated the total space contacted and reports that volume in cubic milliliters. In the second game, ACTIVE measured UE reaching velocity (centimeters per second) and rate of fatigue (percentage of decline) by using the RGB camera to determine the exact location of two yellow targets placed on a tabletop. A blue dot was then placed on the subject’s hand and the game encouraged him to move his hand as quickly as possible between the yellow targets before time ran out. Velocity data was captured up to 30 times per second during each trial. From each of these velocity steps, ACTIVE calculated the minimum, maximum and average velocity of the trial. To determine the player’s rate of fatigue, the average UE movement velocity is calculated for the first and second half of the trial. This method was used to calculate the overall fatigue independently of speed. For example, a subject who moved very slowly would obviously have a slow velocity, but might not show a substantial decrease in velocity from the beginning to the end of the game trial; whereas another subject might start out quickly but could decline in speed substantially within the 30 seconds of a game play test. By comparing the velocities produced within the first and second half of the game play period, the game summarized any decline in velocity observed over time and reported that as fatigue.

Preliminary reliability and validity testing:

Informed consent was obtained from 5 non-ambulatory subjects with a dystrophinopathy (ages 9-36 years) and 5 adult control subjects. The subjects with dystrophinopathy were classified based on their upper extremity abilities using the Brooke upper extremity functional rating scale (Brooke)^29^. The Brooke scale was developed for clinical use in grading general UE function on a 6 point scale, with larger values indicating less function. Each subject completed 3 trials of the FRV and velocity games using each UE to evaluate preliminary reliability between trials. All trials were completed during one study session. Subjects also completed the modified Borg ratings of perceived exertion scale (RPE)^30^^-^ 31. The modified Borg RPE rates how hard the person feels they are working from a score of 6 (no exertion at all) to 20 (maximum exertion). The RPE score was used to compare the subject’s perceived fatigue to the upper extremity fatigue calculated across trials using the Kinect device.

To evaluate validity of the ACTIVE, one non-ambulatory subject with dystrophinopathy was recorded by the Vicon passive marker-based motion analysis system (MC) while he was simultaneously completing 3 trials of ACTIVE velocity test with each upper extremity. In the MC lab, 8 cameras record passive reflection data from markers positioned over the acromion process and on either side of the wrist to define the upper extremity. To enable three dimensional analyses of these data, a cluster of three or more markers, consistent with the method proposed by Cappozzo and colleagues^32^, was placed on each relevant body segment (hand, forearm, upper arm, torso) and also on the chair back for a total of approximately 37 markers. Markered data were filtered (Butterworth dual pass, low pass, 6Hz) and the volume within which the arm moved was calculated as the convex hull of all markers (cubic millimeters) in the Visual 3D analysis program. Velocity of the centroid of one reflective marker, placed on the distal first metacarpal head, was calculated in Matlab as were mean velocities from the first and second half of each game play test.

Ethics statement

This study was approved by the Institutional Review Board at the Research Institute at Nationwide Children’s Hospital. All subjects provided written informed consent to participate.

Statistical analysis

Intraclass correlation coefficients were used to compare sequential trials of the assessments as well as comparing ACTIVE to MC.

## Results

Validity 

Initial validity of the ACTIVE proof-of-concept is supported by its ability to accurately differentiate subjects based on their upper extremity abilities. The ACTIVE discriminately ranked subjects with dystrophinopathy by Brooke scale score and also differentiated them from normal controls (Figure 2) for both the functional reaching volume and velocity tests. Validation against MC demonstrated the stability of the device, but not the absolute accuracy. Data captured simultaneously with the 2 systems showed that the ACTIVE consistently overestimated the subject’s velocity (Mean 20.3% +/- 2.4) (Table 1).

**Figure:**
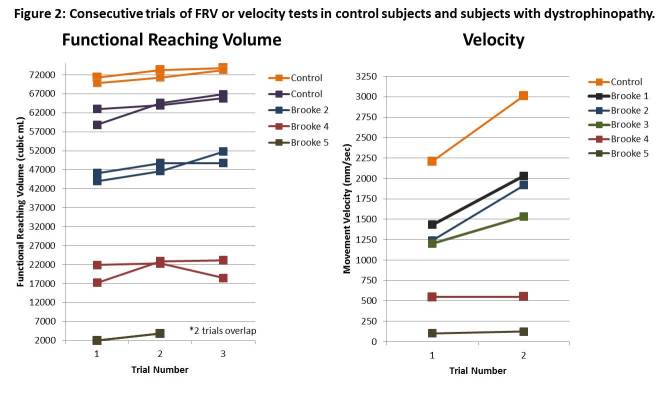

**Figure:**
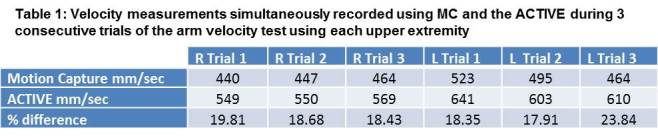

Reliability

When the data are viewed graphically in Figure 2, a learning effect becomes apparent on both the FRV and the velocity test. Preliminary test-retest reliability of the ACTIVE for 2 sequential trials (4 data points were from subjects with dystrophinopathy and 9 data points were from adult healthy controls) was excellent for FRV (ICC=0.986, p<0.001). Similar results were seen with the velocity data with excellent test-retest reliability (ICC=0.963, p<0.001).

Rate of fatigue

Fatigue was studied in a single subject with dystrophinopathy. RPE score increased as his rate of fatigue increased (Table 2).

**Figure:**
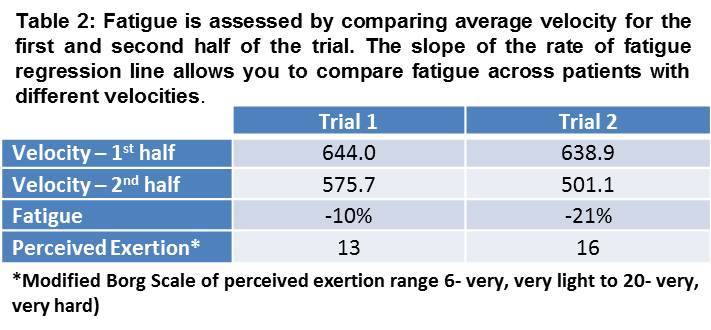

## Discussion

The data from our pilot study with the ACTIVE proof-of-concept device establishes the feasibility of using commercially available gaming technology to create a low-cost, widely accessible, and functional outcome measure for use with children and adults with upper extremity dysfunction across the spectrum of abilities. The cost associated with the Kinect SDK for Windows is under $250, and the device can be purchased conveniently online or in most local retail stores. Our collaboration with a software development company, allowed us to collect and analyze the information collected with the Kinect platform. This post-processing can be completed by any mathematical or statistical department with knowledge regarding interpretation of the xyz-coordinate positional data output provided by the Kinect internal software. This platform has the potential to gather information regarding movement breadth, patterns, speed, and changes in movement variables over time in a variety of conditions affecting the upper extremity. Also, because the output can be analyzed post-examination, the outcome measure could collect any variety of movement patterns of interest for each unique disease progression.

Our ACTIVE proof-of-concept development is novel in several ways. First, knowledge users and patient advocates contributed to the development process. Those involved in the program design included persons with expertise in the clinical care and disease process of individuals with muscular dystrophy, those with understanding of the Kinect platform and its capabilities, personnel with knowledge of mathematical principles necessary to analyze data recorded, and patient advocates and representatives with specific knowledge on the relevance of functional tasks on individual quality of life and independence. Bringing all of these stakeholders together, allowed our team to produce a functional and engaging proof-of-concept with the potential to measure clinically meaningful changes across the lifespan.

To improve upon existing methods to measure FRV, ACTIVE calculated the volume of space available to the participant during the performance of tasks that require cognitive-somatic integration, such as wiping clean a table top or window or reaching for a motivating target (e.g. food, drink of water). This method has the potential to better capture the extent to which the patient can access his environment and better predict his ability to perform functional activities. Current assessments do not capture the unique progression of weakness and loss of function found in dystrophinopathy as they were created for use in other diseases and adopted for use in neuromuscular disease due to the absence of a gold standard. Many of these assessments focus solely or primarily on distal abilities, and are thus incapable of detecting a decline in function until later in the progression of dystrophinopathy 9 10 15 16. Individuals with DMD develop a wide range of compensatory movement patterns in an effort to maintain distal function in the presence of proximal weakness, thereby forestalling functional decline until weakness progresses beyond the point that these compensations suffice 33. These clinical tests of distal function are not capable of quantifying or qualifying these compensatory movements throughout the non-ambulant period^9^. Our pilot study results indicated that the ACTIVE proof-of-concept utilizing the Kinect video-based platform to capture movement data could distinguish between non-ambulatory subjects with dystrophinopathy of differing abilities. Although there appeared to be an initial learning effect, subject performance was consistent across trials within the same testing session. Our device incorporated movements that measured both proximal and distal abilities in one engaging outcome measure.

Lastly, ACTIVE reported velocity and rate of fatigue, or rate of decline in velocity, of UE movement. As described above, velocity of movement is an important component of an individual’s functional independence. Someone may be able to access a portion of their FRV, but if it requires too much time or effort that area may not be utilized frequently. Similarly, if an FRV quadrant is accessible, but induces fatigue quickly, a person may choose not to use that portion of their FRV readily. These variables have implications for functional independence that can provide researchers or clinicians with a full picture of an individual’s change in function over time. ACTIVE differentiated between subjects with dystrophinopathy of differing abilities by velocity of UE movement. Again, test-retest reliability in our pilot study indicated that individual performance was consistent across trials within the same testing session. Our preliminary data on a single patient suggests that the ACTIVE could provide a means for quantifying a subject’s ability to sustain work. Studies of this modality in a larger cohort are underway.

The authors acknowledge that ACTIVE is currently a proof-of-concept and not yet ‘trial ready.’ The purpose of this pilot study was to investigate the feasibility of this type of outcome measure in a population of non-ambulatory individuals with dystrophinopathy. Development of this cutting edge technology is a dynamic process as changes and updates are added immediately as new data are obtained. Future studies will include evaluation of UE abilities in a larger sample to examine ACTIVE’s ability to detect more subtle changes in function versus our current comparison with the broad Brooke levels.

This current proof-of-concept is specifically focused on evaluating UE functional abilities in adolescents and adults with dystrophinopathy. However, this platform has the potential to be used across the lifespan and could have software designed to measure a variety of functional abilities. The engaging gaming aspect of ACTIVE has the potential to increase motivation and understanding of testing directions. Currently participants were encouraged to ‘paint the screen.’ This simple task didn’t require much direction and was somewhat intuitive. Further research is needed to determine the effect of cognitive impairments or behavioral difficulties on cooperation during testing with ACTIVE.

## Summary

This proof-of-concept pilot data demonstrates the feasibility of ACTIVE to capture reproducible upper extremity functional reaching volume, movement velocity, and rate of UE fatigue in individuals with dystrophinopathy, and may discriminate among disease severity when compared to functional rating scales. The adaptation of new gaming technology should allow development of engaging, accurate, and low-cost assessments. This is timely given recent developments in the area of Duchenne muscular dystrophy treatment, where trials are underway or planned for exon-skipping, stop codon read-through, and gene therapies 34, but this technology also holds promise for the assessment of many other neurologic and musculoskeletal disorders.

## Competing Interests

Kevin M. Flanigan is an editor of PLOS Currents Muscular Dystrophy but was not involved in the evaluation of this article. The authors have declared that no other competing interests exist.
